# Determining the Contribution of Epidermal Cell Shape to Petal Wettability Using Isogenic Antirrhinum Lines

**DOI:** 10.1371/journal.pone.0017576

**Published:** 2011-03-10

**Authors:** Heather M. Whitney, Rosa Poetes, Ullrich Steiner, Lars Chittka, Beverley J. Glover

**Affiliations:** 1 Department of Plant Sciences, University of Cambridge, Cambridge, United Kingdom; 2 School of Biological Sciences, University of Bristol, Bristol, United Kingdom; 3 Cavendish Laboratory, Department of Physics, University of Cambridge, Cambridge, United Kingdom; 4 School of Biological and Chemical Sciences, Queen Mary, University of London, London, United Kingdom; Trinity College Dublin, Ireland

## Abstract

The petal epidermis acts not only as a barrier to the outside world but also as a point of interaction between the flower and potential pollinators. The presence of conical petal epidermal cells has previously been shown to influence the attractiveness of the flower to pollinating insects. Using Antirrhinum isogenic lines differing only in the presence of a single epidermal structure, conical cells, we were able to investigate how the structure of the epidermis influences petal wettability by measuring the surface contact angle of water drops. Conical cells have a significant impact on how water is retained on the flower surface, which may have indirect consequences for pollinator behaviour. We discuss how the petal epidermis is a highly multifunctional one and how a battery of methods, including the use of isogenic lines, is required to untangle the impacts of specific epidermal properties in an ecological context.

## Introduction

The epidermis of a plant acts as a barrier to the outside world, providing a waterproof layer that prevents dehydration of internal tissues. The majority of plant epidermal surfaces are composed of essentially flat cells. The occurrence of protruding cells, particularly trichomes (hairs) and papillae (single cells in the shape of cones), is associated with specific functions. For example, trichomes may be involved in deterring predators as well as moderating leaf boundary layer, and have also been found to influence the degree to which water is retained on the plant epidermis – its wettability [1]. Previous studies have indicated that approximately 80% of plant species analysed have petal epidermal surfaces composed exclusively, or almost exclusively, of conical cells [2]. The restriction of conical cells to the petal epidermis, and the frequency with which they are found on petals, has led several authors to conclude that they must function to enhance the attractiveness of the corolla to pollinating animals. There has been considerable debate as to how conical cells might function to increase floral attractiveness [2]–[7].

**Figure 1:**
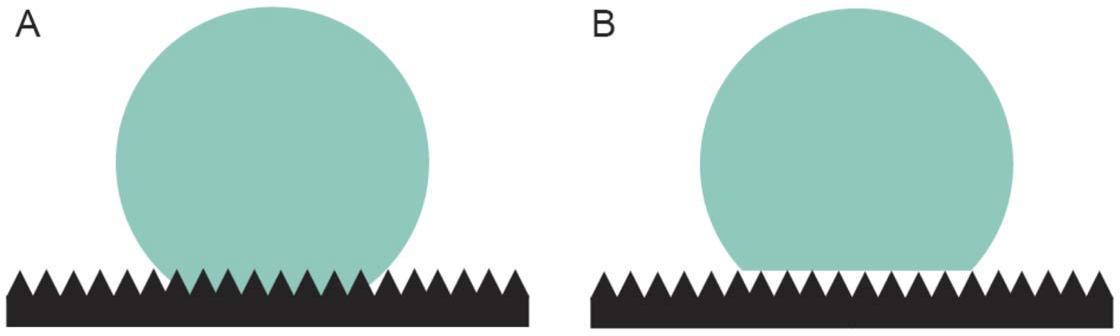
Diagram illustrating wettability behaviour of water on a rough surface. **A**. Wenzel wetting, where the water is in close contact with the surface. **B.** Cassie-Baxter wetting where air is trapped between parts of the surface and the drop.

It is also possible that petal cell shape affects floral surface wettability. Structures on the plant surface and surface chemistry can both have a significant effect on hydrophobicity or hydrophilicity [1], [8], [9]. The behaviour of surface water on a rough surface such as the plant epidermis was established by Wenzel [10] and Cassie and Baxter [11]. In ‘Wenzel wetting’ the water is in close contact with the surface (Figure 1A) while in ‘Cassie-Baxter wetting’ air is trapped between parts of the surface and the drop (Figure 1B). Due to the presence of air-pockets under the droplet in Cassie-Baxter wetting, the water has less physical contact with the surface. In this case the drop has a very small contact angle hysteresis and rolls easily off the surface. In this case, the surface is superhydrophobic [8], [12].

Such superhydrophobicity was previously observed on the leaves of the Sacred Lotus, *Nelumbo nucifera*, where extracellular conical wax extrusions present on these leaves reduce the wettability of the surface, allowing water droplets to bead and roll off [13], [14]. This adaptation is clearly important for an aquatic plant, but it may also be of significance in maintaining leaves and petals of other species free from waterlogging. A consequence of the lack of wettability of the Lotus leaf is its “self-cleaning” properties. Water droplets rolling off the leaf remove particles of dirt, generating a clean surface, an effect known as “the Lotus effect”, which has provided the inspiration for biomimetic applications [13]. A lack of wettability on the leaf surface is thought to be a distinct advantage for several other reasons including that water reduces photosynthetic gas exchange, may promote pathogen infection and may enhance pollutant deposition [15], [16].

In the case of flowers, wettability could influence pollinator preference. The presence of conical cells has already been shown to impact on pollinator choices [3]–[6]. If the conical cells of petals generate a lack of wettability similar to that found in the Lotus effect, this may have a range of adaptive consequences, including potentially impacting on the ability of an insect to successfully grip a flower, a factor that has been shown to influence pollinator preferences [6]. The potential self-cleaning properties could also have an impact on the presence of scent marks, colour-obscuring dust, and bacterial cells and fungal spores, all of which could affect pollinator choice and contribute to general plant health [17]–[19].

However, the wettability properties of the plant surface have so far only been studied either through biomimetics or through comparing the structural and chemical differences between the epidermal surfaces of different plant species from different habitats [14], [15], [20]–[22]. Biomimetic methods can precisely determine how individual surface features influence wettability, and comparisons of different species determine how overall surface may influence plant ecology. This creates a difficulty in directly determining the ecological impact of specific surface wettabilites due to the range of interspecies difference in surface properties. The ability to manipulate individual components of the plant surface, and so directly impact on surface wettability, would be an invaluable tool to investigate the ecological relevance of this physical property. This can be achieved by the use of genetically modified or mutant lines.

**Figure 2:**
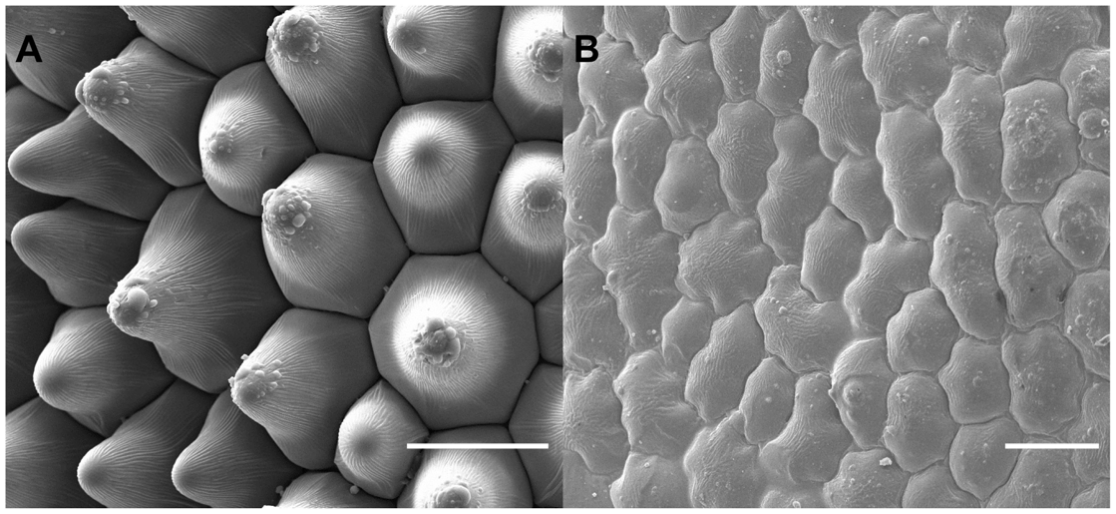
Conical-celled and flat-celled petal surfaces. **A.** Scanning Electron Microscope image (SEM) of wild-type Antirrhinum petal. **B.** SEM of *mixta* mutant Antirrhinum petal.

One mutant line that has been invaluable in the study of the function of the plant epidermis in plant-insect interactions is the *mixta* mutant of Antirrhinum. The *mixta* mutant lacks conical papillate petal cells, and instead produces flat petal epidermal cells, more similar to leaf epidermal cells (Figure 2). The mutant is a lesion in a single gene and has no other consequences for plant phenotype, including in the composition of cuticular waxes [3], [23]. The use of isogenic lines differing only in the *MIXTA* locus and therefore only in the shape of the petal epidermal cells allows accurate and sensitive dissection of the function of these specialised cells. Using these lines we have previously shown that conical cells increase fruit set and reduce pre-landing and post-landing rejection of flowers by bees [3], [4].

In this paper we consider the effects of conical cells on petal wettability. This is the first study to test the impact on plant surface wettability of changes in the plant epidermis due to a single gene, and to provide a model system in which the ecological importance of this property can be tested. Since bees can distinguish between the different colours of wild type and *mixta* flowers, and can learn to associate those colour differences with different rewards [5], [24], they could be using them as a cue associated with some other physical property of the flower. If cell shape significantly affects the wettability, and pollinators exhibit discrimination between flowers of different dryness, then this physical effect could also explain the preference of bees for conical-celled flowers.

Here we show that petal cell shape has a significant influence on floral wettability. Conical cells have been shown to be multifunctional, and thus we conclude that the ability of conical cells to influence floral wettability could be one of the factors by which conical petal cells enhance plant fitness. We conclude that the use of isogenic lines is a powerful tool for the study of the ecological importance of plant surface wettability.

## Materials and Methods

### Plant lines and growth conditions

Two lines of Antirrhinum plants were used, varying in petal cell shape. The isolation of the isogenic wild-type (*Mx^+^*), and *mixta* (*mx^−^*), lines is described in [3]. These lines have been maintained by self-pollination since 1995 so genetic variation between individuals is almost absent. The *mixta* mutant has flat petal epidermal cells [23].

Six plants of each of the Antirrhinum lines were grown under greenhouse conditions at 23°C in 4-inch pots in Levington's (UK) M3 compost. During the growth period plants received supplemental lighting from 400 Osram (Osram, München, Germany) lamps on a 16 hr light/8 hr dark photoperiod. Inflorescences with fully opened flowers were selected from each plant as they became available, and flowers within the inflorescence further selected to avoid flowers with petal surface damage or irregularities.

### The effect of petal cell shape on wettability

Surface wettability can be mostly characterised by two measurable quantities, the static contact angle of the water droplet and the contact angle hysteresis. The wettability of the fresh petals of the conical-celled wild type and flat-celled *mixta* mutant was determined by measuring the surface contact angle of single water drops as they were extruded onto the surface via a syringe needle (the advancing contact angle) or were removed (receding contact angle). The difference between the advancing and receding contact angles defines the contact angle hysteresis. If part of a droplet of water remained after attempted removal, a measurement of the static contact angle was taken.

**Figure 3:**
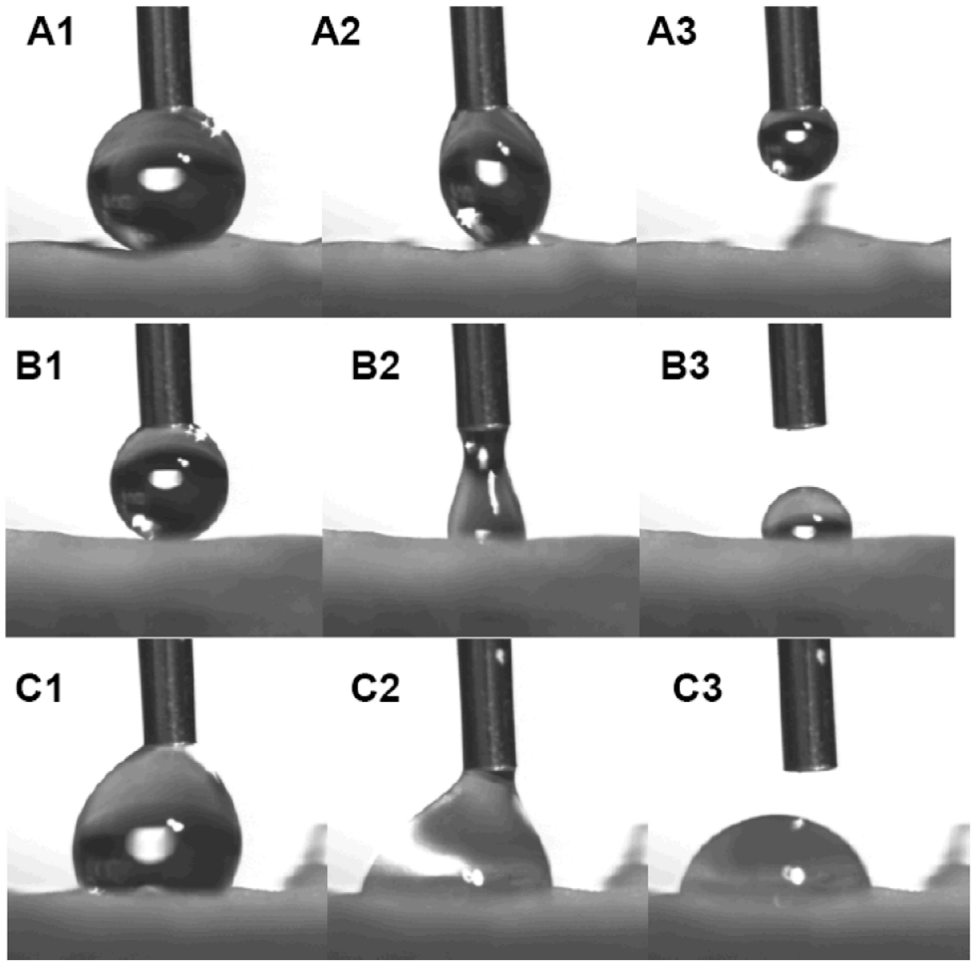
Measurement of floral surface wettability. **A1**. Advancing angle of drop on surface showing Cassie-Baxter wetting. **A2**. Receding angle of drop showing Cassie-Baxter wetting. **A3**. Ease of drop removal on a surface showing Cassie-Baxter wetting. **B1**. Advancing angle of drop on surface showing Partial Cassie-Baxter wetting. **B2**. Receding angle of drop showing Partial Cassie-Baxter wetting. **B3**. Drop removal on a surface showing Partial Cassie-Baxter wetting, showing that while initial removal is similar to perfect Cassie-Baxter wetting, a localized point remains. **C1**. Advancing angle of drop on surface showing Wenzel wetting. **C2**. Receding angle of drop showing Wenzel wetting. **C3**. Attempted drop removal on a surface showing Wenzel wetting.

Two samples were cut from each of the lobes of 10 individual flowers from each line. All samples were laid flat and attached to a glass microscope slide by means of double-sided tape. A visual inspection of the sample confirmed that sample preparation had not damaged the petal surface, and any samples showing damage or irregularities were discarded. Using the methods detailed in [25], measurements from were each sample were obtained using a contact goniometer (KSV CAM 200) equipped with a digital image acquisition system and an automatic liquid dispenser. Individual water drops were dispensed and then removed from the petal surface. If the sample was large enough, a second measurement was subsequently taken on a separate region of the sample. Images of the water drop as it advanced and receded across the petal surface were taken (Figure 3 shows images of advancing and receding water drops on the petals of both wild type and *mixta* lines). The contact angles were determined from these side-on images of the drops using a numerical fitting algorithm [25].

## Results

### Petal cell shape has a significant effect on petal wettability

The wetting behaviours showed by the Antirrhinum petals could be categorised into three types of wetting behaviour; (**A**) Wenzel wetting (where the water is in close contact with the surface), (**B**) Cassie-Baxter wetting (where air is trapped between parts of the surface and the drop) and (**C**) Partial Cassie-Baxter wetting [10], [11]. This third, intermediate type of wetting behaviour was found on the wild-type petals, where the drop was found to have very small contact angle hysteresis, but part of a droplet of water remained after attempted removal. Examples of all three types of wetting on Antirrhinum petal surfaces are shown in Figure 3.

Of the 31 measurements made of the conical celled wild-type petals, drop behaviour corresponded 4 times to complete Cassie-Baxter wetting, 7 times to partial Cassie-Baxter wetting and 20 times to Wenzel wetting (see Table 1 for details and properties of the different wettability categories). Of 14 measurements made of the flat-celled *mixta* mutant, all showed Wenzel wetting. If the mutants showed the same wetting as the wild types, the probability of an individual mutant sample showing Wenzel wetting would be 0.645 and the probability of 14 mutant samples (out of 14) showing Wenzel wetting can therefore be calculated as 0.002 (exact value from binomial test). Mutants therefore have significantly different wetting properties to wild type flowers.

**Table 1:** Designated wettability criteria and occurrence in Antirrhinum wild type (*Mx^+^*) and *mixta* (*mx^−^*) lines.

	Perfect Cassie-Baxter wetting	Partial Cassie-Baxter wetting	Wenzel wetting
	Picture Sequence A	Picture Sequence B	Picture Sequence C
Ease of drop removal	Very easy	Easy but with one localised point remaining	Not easy
Number of examples in wild-type Antirrhinum flowers	4	7	20
	Average angle when drop was:	Average angle when drop was:	Average angle when drop was:
	advancing (148±6)	advancing (140±18)	advancing (135±17)
	receding (109±22)	receding (91±16)	receding (73±19)
Number of examples in *mixta* Antirrhinum flowers	0	0	14
			Average angle when drop was:
			advancing (120±14)
			receding (74±14)

Picture sequences refer to Figure 3.

## Discussion

We find that, if studied independently of surface chemistry by using an isogenic mutant line, cell shape does play an important role in determining petal wettability. The presence of conical cells renders the surface weakly superhydrophobic. This weak superhydrophobicity is not present for the *mixta* mutant, which lacks the conical-celled surface corrugation. Generally, if they had an identical cuticular composition, flat-celled petals would therefore be more wettable than conical-celled flowers, whether of the same or different species. This finding is in line with studies by Barthlott and Neinhuis [13], [14], which showed that the papillate leaf cells of the Sacred Lotus *N. nucifera* were significantly less wettable than ordinary flat leaf cells. They concluded that this lack of wettability helped the plant to prevent waterlogging in its aquatic habitat, and showed that the formation of beads of water caused dirt to be more easily removed from the leaf.

The conical cells found on Antirrhinum flowers produce a much less robust hydrophobicity than that shown on *N. nucifera* as the exact contact angle was strongly influenced by very local petal surface properties. As well as Wenzel and Cassie-Baxter wetting, a third type of wetting was also found on the wild type flowers. While the division between Wenzel and Cassie-Baxter wetting is usually very distinct, surfaces can display mixtures of these two behaviours. This is the case for surfaces that are close to the crossover between these two regimes, for which both wetting types have similar free energies (weakly superhydrophobic surfaces). On natural surfaces, even small amounts of structural damage can induce Wenzel wetting on surfaces that otherwise have Cassie-Baxter properties. This suggests that even small regions of damage caused by pathogens or insects or small amounts of contamination, such as grains of pollen, could be enough to seriously perturb the degree of hydrophobicity. The observed weak superhydrophobicity might therefore have a self-cleaning function for dewdrops that form directly on the petal surfaces, but probably not for impacting drops (such as rain).

The self-cleaning effect observed for Lotus leaves could therefore explain the prevalence of conical cells on Angiosperm petals. Petals are exposed to dust, dirt and pollen, which might interfere with their display and their attraction of pollinators. They are also exposed to pathogenic bacteria and fungal spores. Ability to remove all of these contaminants by self-cleaning could significantly enhance pollination success, although each contaminant will be differently influenced by the surface, depending on viscosity. The self-cleaning properties observed in petals were much less robust than those found on Lotus leaves. This possibly arises from the difficult handling of the petals in the contact angle goniometer. However, lotus-like surface features (micrometre-sized wax crystals on surfaces) could potentially interfere with other floral roles, such as pollinator handling and colour display. Conical cells, on the other hand, not only do not interfere with other floral roles, but actually enhance both the visual display and ease of pollinator handling while providing a degree of hydrophobicity [6], [7], [23] and therefore, though less robust than the lotus-like surface features, may be optimal for both display and protection. Analysis of the distribution of conical petal cells with relation to habitat, dew and rainfall and pollinator type would allow discrimination between some of these possibilities.

As mentioned before, the petal epidermis is highly multifunctional. Individual floral features, for example conical cells, have been implicated in a wide range of floral properties. Due to the *mixta* Antirrhinum line, the various impacts of conical cells have begun to be untangled, and they have been found to impact directly on floral colour, floral temperature, flower shape, pollinator grip and now flower wettability (this study). How much each of these factors contributes to the maintenance of the presence of conical cells in such a high proportion of flowering plants, how much the production of conical cells costs, and the trade-off between these factors will be the subject of on going ecological studies both to establish the contributions of these cells in different situations and to determine why a feature that appears to be useful in such a variety of ways is absent from a proportion of Angiosperm species..

A similar approach could be used to determine the strengths of the hypotheses regarding wettability of leaves. Hydrophobicity in leaves is thought to be maintained due to a number of advantages, including that a layer of water reduces photosynthetic gas exchange, promotes pathogen infection and enhances pollutant deposition [15], [16]. Using isogenic lines that vary in wettability traits within ecologically relevant contexts could test each of these ideas.

In conclusion, we have shown that petal cell shape does significantly influence the physical properties of petals. The commonly found conical petal epidermal cells make a great deal of difference to wettability. Thus, besides providing visual and tactile cues to pollinating animals, conical petal cells may also act to prevent waterlogging and to optimise shedding of dirt and pathogens. Conical cells have been found to be multifunctional, and these many roles may explain the prevalence of the conical petal cell form in the flowering plants.
